# Clinical and Epidemiological Correlates of Task-Specific Dystonia in a Large Cohort of Brazilian Music Players

**DOI:** 10.3389/fneur.2017.00073

**Published:** 2017-03-06

**Authors:** Rita C. Moura, Patrícia Maria de Carvalho Aguiar, Graziela Bortz, Henrique Ballalai Ferraz

**Affiliations:** ^1^Movement Disorders Unit, Department of Neurology and Neurosurgery, Universidade Federal de São Paulo (UNIFESP)/Escola Paulista de Medicina, São Paulo, Brazil; ^2^Hospital Israelita Albert Einstein (HIAE), São Paulo, Brazil; ^3^Instituto de Arte, Universidade Estadual Paulista (UNESP), São Paulo, Brazil

**Keywords:** dystonia, musician’s task-specific dystonia, motor control, occupational health, movement disorders in musicians

## Abstract

Musician’s dystonia is a task-specific dystonia (TSD) worldwide disabling disorder, and most of the affected individuals may have severe difficulty to play their instrument. Many professional music players may have to quit working as a player. The objective of the present study was to evaluate the clinical characteristics and frequency of TSD in Brazilian music players and to promote awareness of this condition among musicians. We visited orchestras and music schools delivering lectures on TSD and about the scope of our survey. Musicians were invited to answer a questionnaire, and those with possible neurological dysfunction associated with musical performance were recorded by video while playing the instrument. We visited 51 orchestras and music schools in 19 Brazilian cities between March 2013 and March 2015. We collected 2,232 questionnaires, and 72 subjects with suspicion of dystonia were video recorded during specific tasks and evaluated regarding motor impairment. Forty-nine individuals (2.2%) were diagnosed as having TSD (mean age 36.4 years; 92% male). The instruments most associated with TSD were acoustic guitar (36.7%) and brass instruments (30.6%). We concluded that Brazilian TSD music players are mainly male, classical music professionals, around 30 years of age, with arms, hands, or oromandibular muscles affected. TSD is a neurological condition that can impair musical performance and should receive more attention from musicians, teachers, and health professionals.

## Introduction

Focal dystonia in music players is a task-specific dystonia (TSD) triggered by a specific motor act. It is localized and triggered by simultaneous activities of agonist and antagonist muscles, inducing abnormal movements or postures, particularly in the hands, fingers, face (embouchure), neck (torticollis), or tongue, usually without pain (1–3). Its primary cause is unknown, but some findings suggest there are abnormalities of sensorimotor integration in specific, refined, high-performance tasks (4).

Musician’s dystonia is difficult to diagnose, assess, and classify, and it may be underdiagnosed. Symptoms of TSD in musicians often appear between the ages of 30 and 40 years. The first signs of dystonia may be slips, technical mistakes or apparent lack of preparation in excerpts previously performed without issues and, within months, the performance problems progressively intensify. Generally, there is no associated pain, and dystonia is absent in activities other than playing the musical instrument (5–9).

Several studies have focused on determining the prevalence of dystonia among music players and showed considerable variation (0.5–13%). In Brazil, dystonia is not an object of discussion or interest among musicians, and many cases are diagnosed too late due to the lack of knowledge. To date, no epidemiological data are available to define the profile of music players with dystonia in the country. Awareness of this condition and early diagnosis are extremely important in order to adopt preventive measures and to offer adequate treatment.

This study aimed to assess the frequency and clinical profile of musician’s TSD in Brazil as well as to promote awareness about this condition among them.

## Materials and Methods

Between March 2013 and March 2014, we conducted a nationwide study, visiting 51 representative professional orchestras and music schools in 19 cities of Brazil. We included all music players (students or professionals) attending our talks and who agreed to fill in a questionnaire. Those excluded were individuals presenting with another neurological condition or NTSD, and those who reported a loss of movement control but were unwilling to undergo the neurological evaluation. The present study was approved by the Ethical Committee on Research of UNIFESP/EPM, and informed written consent was obtained from all subjects who were told about the purpose of the study and of being surveyed on motor problems while performing musical instruments.

At each orchestra and music school, we delivered a lecture to explain the goals of the study, the clinical characteristics of dystonia and of musician’s TSD, including possible therapeutic approaches. Subsequently, we distributed a specific self-administered questionnaire to obtain frequency, clinical and study/work routine information.

The questionnaire consisted of the following information: age; gender; course of study; instrument; age when started to play the instrument; number of daily hours of playing (studying and working); whether they were students or professional musicians; whether they played classical or popular music; whether they played in an orchestra or in a band; handedness; use of the hands in other activities; presence of pain in the affected segment; intensity of pain; presence or absence of other conditions (pain was evaluated only considering the subjective perception of the individual—no visual scale was used); sensations during practice, weakness of the affected segment of the body; whether the individual received a previous diagnosis and what that was; whether there was a previous treatment proposed; and family history of dystonia. This questionnaire has not been published or previously validated.

When the questionnaire indicated a motor dysfunction or the music player directly reported a problem in musical execution, he or she was invited to be video recorded. During this recording, the individual played two-octave musical scales at two different speeds and performed musical excerpts likely to show the signs of motor disorder. The music player also underwent a positional tremor assessment; those with an upper limb motor dysfunction performed a writing task, and those with face or tongue dysfunction read a text out loud. Musical performance and the reading/writing tasks were videotaped in 3-min segments (at minimum).

Since dystonia can be associated with anxiety, the participants were evaluated with the Hamilton Anxiety Scale (2, 6, 10–14). The severity of dystonia was evaluated using the Tubiana and Champagne scale (15–17). Both scales were administered before the video recording. The video recording and the application of the anxiety and dystonia severity scales were done by a physiotherapist specialized in musician’s movement disorders.

The videos were analyzed independently by two neurologists skilled in movement disorders (PMCA and HBF), and the diagnosis was then reached by consensus.

We diagnosed dystonia following the criteria of Albanese et al. (1). We did not use electromyography in any of the individuals of this sample. We classified the evaluated musicians into one of the following six categories:

– Definite dystonia (DTSD): presence of undoubted visible dystonic movement (sustained or intermittent muscle contractions causing abnormal movements or postures) while playing an instrument.
– Probable dystonia (PrTSD): a compatible history, observed loss of control during performance in video, but the postures or movements were not sufficient in intensity or consistency to justify the classification as DTSD. Sometimes, although there were noticeable performance difficulties, the supposedly dystonic muscle was hidden from the examiner view by the instrument or anatomically, particularly in the cases with lips or tongue involvement.
– Possible dystonia (PoTSD): a compatible history, but abnormal movements and/or a compromised performance were not detected in the video (18–20).
– Dystonia not associated with musical practice (DNAMP): undoubted dystonic movements in daily activities other than playing an instrument. In some cases, playing even stopped the dystonic movements.
– Absence of dystonia: incompatible history and/or observation of a different condition related to movement, e.g., tremors.

In the statistical analysis, only the musicians classified with DTSD or PrTSD and those whose dystonia was exclusively associated with musical practice were considered. Comparisons were made between dystonic (DTSD or PrTSD) and non-dystonic music players. For categorical variables, chi-square or Fisher’s exact tests were applied. For numerical variables, Student’s *t*-test were used, a *p* value <0.05 was considered statistically significant.

## Results

We obtained 2,233 answered questionnaires, with 1,915 music players reporting normal motor control during musical practice. The remaining 318 reported a lack of motor control during the performance. Eighty-two of them underwent the specific evaluation, although 10 did not agree to be videotaped. Thus, 72 music players were video recorded, and we classified 42 (58.3%) as having DTSD; 7 (9.7%) PrTSD; 7 (9.7%) PoTSD; and 15 (21%) as non-dystonic (including one who presented with tremors associated with musical practice but without characterizing dystonia) and 1 NAMPD.

For the statistical analysis, we considered two groups: (a) dystonic music players (*n* = 49), including those classified as DTSD and PrTSD, and (b) non-dystonic music players (*n* = 1,930), including the 1,915 who did not report any motor dysfunction and the 15 who, after evaluation, were diagnosed as non-dystonic. The remaining 254 music players were excluded: those classified as PoTSD (7 individuals) or DNAMP (1 individual), musicians who did not consent to being recorded in the video (10 individuals) and music players who reported some sort of motor dysfunction (236 individuals) but did not take part in the individual evaluation (Table 1).

**Table 1:** **Epidemiologic characteristics**.

Parameter		DM	NDM	*p*

		*N* (%)	*N* (%)	

		49 (2.2%)	1,930 (86.5%)	
Age		36.4 ± 12.2	26.5 ± 10.7	5.9532E−07*
Gender	Male/female	45 (92)/4 (8)	130 (68.3)/608 (31.7)	0.000357*
Handedness	Right-handed	42 (85.7)	1,648 (87.2)	0.753578
	Left-handed	5 (10.2)	217 (11.5)	1
	Ambidextrous	2 (4.1)	25 (1.3)	0.130707
Musical genre	Classical	35 (71.4)	1,240 (64.2)	0.299852
	Pop music	3 (6.1)	394 (20.5)	0.013619*
	Classical and pop	11 (22.4)	296 (15.3)	0.174448
Player occupation	Professional	30 (61.2)	550 (29.3)	1.52E−06*
	Student	16 (32.7)	1,251 (66.6)	7.36E−07*
	Semi-professional	3 (6.1)	76 (4.0)	0.470024
Other occupation		30 (61.2)	574 (29.7)	2.29E−06*
Other occupation/upper limbs		22 (44.9)	15 (0.78)	0.0
Years of musical practice		21.5 ± 13.1	12.5 ± 10.0	1.815E−05*
Daily music study (h)		2.8 ± 1.7	3.1 ± 1.5	0.2177
Daily music work (h)		4.57 ± 2.9	3.84 ± 1.9	0.1644
Family history		4 (13.3)	1 (12.5)	0.9505

*DM, dystonic musicians; NDM, non-dystonic musicians*.

*MMSS—other occupation using upper limbs*.

*Denotes that there was a statistical significance of <0.05*.

There were 580 professional music players and 1,346 music students or semi-professional performers. Among professional players, there were 30 TSD individuals (5.2%) and 19 (1.4%) among students/semi-professional players. The different frequency of dystonia in both groups was statistically significant (*p* < 0.01). Total daily hours dedicated to work or study was not different in both groups. However, the total of years dedicated to music was different (mean of 27.2 years in professionals and 12.2 years in students/semi-professionals).

Of the 1,979 musicians included in the analysis, gender was not identified in 14 of the received questionnaires; from the 1,935 musicians, 612 (31.1%) were women, and 1,353 (68.9%) were men. Thirteen of the dystonic musicians showed signs of anxiety: 7 (14.3%) mild, 2 (4%) medium, and 4 (8.1%) severe.

Musicians rated the severity of dystonia as follows: 7 (14.3%) reported as incapable of playing; 7 (14.2%) played without proficiency; 6 (12.2%) showed slowness when playing; 6 (12.2%) could play easy pieces; and 20 (40.9%) were almost at a normal level of performance and 1 did not answer (Table 2).

**Table 2:** **Clinical characteristics**.

Parameter	DM	NDM	*p*

	*N* (%)	*N* (%)	

	49 (2.2%)	1,930 (86.5%)	
Fatigue	33 (67.3)	1,008 (52.2)	0.0363381*
Pain	18 (36.7)	717 (37.2)	0.95259
Musculoskeletal disorders	15 (30.6)	386 (18.4)	0.031164*
Abnormal sensitivity	11 (22.4)	429 (22.2)	0.970692
Tremor (all)	33 (67.3)	3 (0.15)	0
Dystonic tremor	15 (30.6)	–	–
Postural tremor	16 (32.6)	3 (0.3)	0
Postural/dystonic tremor	2 (4.0)	–	–
Musicians with diagnosis	26 (61.9)	14 (56.7)	0.711326
Seek for medical care	31 (63.3)	5 (0.3)	5.53E−23*
Duration of dystonia (in months)	71.1 ± 86.9	–	–
Age of onset of dystonia (in months)	30.8 ± 10.8	–	–
Anxiety	13 (27.1)	6 (46.2)	0.187806

*DM, dystonic musicians; NDM, non-dystonic musicians*.

*Denotes that there was a statistical significance of <0.05*.

The number of dystonic music players and their respective instruments were: 18 guitar, 6 trombone, 5 piano, 3 French horn, 3 tuba, 2 clarinet, 2 bassoon, 2 flute, 2 saxophone, 2 cello, 1 recorder, 1 trumpet, 1 viola, and 1 violin (Table 3).

**Table 3:** **Instrument families**.

Instrument	DM	NDM	*p*

	*N* (%)	*N* (%)	

	49 (2.2%)	1,930 (38.5%)	
Winds	22 (44.9)	518 (26.8)	0.00507*
Brass	15 (30.6)	276 (14.3)	0.003507*
Woodwind	7 (14.30)	242 (12.5)	0.066373
Piano	5 (10.2)	198 (10.3)	1
Guitar	18 (36.75)	241 (12.5)	1.9E−05*
Bowed string (i.e., violin, viola, and cello)	4 (8.15)	617 (32)	0.000138*
Others	0	345 (17.8)	–
Uninformed	0	11 (0.6)	–

*DM, dystonic musicians; NDM, non-dystonic musicians*.

*Denotes that there was a statistical significance of <0.05*.

Of the 22 wind players, 16 had affected embouchure (4 trombone, 3 French horn, 2 tuba, 1 saxophone, 1 clarinet, 1 flute, and 1 trumpet) and tongue (2 trombone and 1 tuba), 1 in the right upper limb (clarinet), 4 in left upper limbs (1 bassoon, 1 flute, 1 recorder, and 1 saxophone), and 1 in both (bassoon). Of the 18 guitarists, 12 were affected in the right hand, 4 in the left hand, and 2 bilaterally. Of the four bowed string instruments players, two were affected in the left hand (violin and viola), one in the right hand (cello), and one in the left arm (cello). Of the piano players, four were affected in the right hand and one in the left hand (Table 3).

Out of 33 music players with upper limb dystonia, 18 were affected in the right hand, 11 in the left hand, 1 in the left arm, and 3 bilaterally. Of these, 29 (87.9%) were right-handed, 3 (9.1%) left-handed, and 1 (3.0%) ambidextrous.

Nine of these music players with upper limb dystonia (five guitarists, one cellist, one clarinetist, and two pianists) developed writer’s cramp after the onset of dystonia; all were right-handed with and dystonia affected the right hand.

Tremor was present in 29 (59.1%) of the 49 dystonic. The same body part affected by dystonia manifested tremor in 85.0% of the cases.

## Discussion

To our knowledge, this is the first comprehensive study of TSD in Brazilian music players and the largest in Latin America.

The frequency of musician’s dystonia (2.2%) in this sample was similar to those reported by other surveys (9, 17). The estimated prevalence varies greatly, depending on the type of study, region, population, types of instrument, or musicians interrupting their musical activities without undergoing an adequate evaluation by a specialist (5–9, 12, 15, 17, 18, 21–24). Our frequency rate could potentially be an underestimate. First, some of the musicians had been away from their activities and could not be contacted by us. We could speculate that some of these cases could be related to dystonia. Second, we perceived considerable concern about and even rejection of the opportunity to undergo an evaluation; being identified by peers and employers as ill was seen as very negative, posing a threat to their job or even their career.

Among the dystonic musicians (DMs), the mean age at diagnosis was similar to that reported by other studies, which is around 33–36 years of age. In general, it takes a long period before they can obtain a correct diagnosis (2, 5–9, 11, 12, 19, 21, 25, 26). Most of the DMs were unaware they were dystonic; only 18 were previously diagnosed as dystonic, although 31 (63.3%) had undergone medical care for the condition.

Dystonic musicians had more years of studying and practicing the instrument than non-dystonic musicians (NDMs) although the number of hours of study and work was the same in both groups. We may infer that a longer duration of practicing may be a risk to developing dystonia. On the other hand, we can also suppose that the earlier the individual begins to practice the greater the risk of developing dystonia.

Professional music players had a higher frequency of dystonia as compared to students or semi-professional music players indicating that more years of practice may be a significant risk factor.

Nine DMs reported difficulties in writing after the onset of dystonia, indicating that TSD may progress affecting other tasks.

Besides the total duration of practice, our results show that the male gender is also a risk factor for dystonia, which is in accordance with previous reports (2, 6, 13, 21). Male-to-female ratios for dystonia are 4:1 or 2:1 (2, 6, 8, 9, 12, 13, 17, 21, 22, 24). In our study, the ratio was far higher, at 10:1. These numbers could be reflecting the fact that Brazil has twice as many male music players as females. It is unclear why men are more prone to dystonia. According to Altenmüller, this could be due to hormonal differences, psychological attitudes or even different stress factors between men and women (22, 23). Going further, it is possible that women are more prone to painful sensations and more readily subject themselves to medical care, addressing any potential pathological processes earlier.

In classical music, high levels of performance are generally expected from the musician, and mistakes are usually unacceptable. This context may lead to unmeasured fastidiousness and to physical and emotional overload usually sustained for long periods (3, 5, 7). Our findings showed that popular music musicians were two and a half times less prone to dystonia than classical musicians. Although popular music can also require a highly technical performance, extended periods of study and sophisticated and complex motor adjustments, the expectations and the demands on the musicians are usually smaller in classical music (21, 27).

We could also observe that music players who practised other activities concurrently with their musical practice were more prone to developing dystonia. We investigated this to establish whether dystonia was related to other activities that also demand physical strain from the upper limbs, particularly considering the cases with dystonia in these parts of the body. Besides orchestral playing and daily practice, the other activities included conducting, library duties, teaching, researching, studying, and others that were less frequently reported. We can suppose that these other tasks may overload the upper limbs, expressing dystonic movements in other activities after the onset of TSD (2, 5–7, 27).

The instruments played by the majority of the dystonic were those that demand greater ability from the right hand (mainly guitarists) or high physical pressure needed for the control of amplitude and frequency of vibration in the facial muscles/embouchure (trombone and tuba), which is in accordance with studies that show a higher incidence of dystonia with instruments that demand greater sensorimotor precision (5, 9, 12, 17, 24, 28, 29). Most of the affected musicians were right-handed. Our results showed that the sites affected by dystonia coincided in 60% of the cases with the dominant laterality of the individual.

Interestingly unlike previous studies, we were unable to show a correlation between dystonia and family history (1–4, 18, 19, 21, 26, 27).

Fatigue was consistent (over 50%) across all groups. It was more common in dystonic wind players (particularly brass instruments) and in dystonic guitarists than in other instrument families. Fatigue is a symptom present throughout the performance of DMs (1, 5, 22). It was consistent (over 50%) across all groups, being more common in dystonic wind players and guitarists.

Several studies have reported that dystonia was not associated with pain, although pain could be a symptom that precedes the development of dystonia (5, 11, 12, 15, 19, 23, 27). We did not observe a different frequency of pain between dystonic and NDMs. Pain was usually not associated with dystonia, but rather with musical practice.

Previous musculoskeletal illnesses were associated with every group diagnosed with dystonia when compared to NDMs. Previous studies have shown that muscular injuries could precede or trigger the development of focal dystonia in musicians susceptible to these movement disorders (2, 5, 6, 13, 26).

The presence of tremors is a frequent symptom associated with dystonia in over 50% of the cases (2, 5, 8, 17, 22). We observed a higher incidence of tremor in dystonic groups (67.3%) and, in most of the cases, the tremor was present in the limb affected by dystonia.

We found that musicians who made themselves available for the evaluation, believing that something was not right, misevaluated their performance, even when diagnosed as dystonic. This could be due to an observed tendency that music players usually minimize psychological and biological problems (11, 13, 23, 26).

Brazilian music players are subject to a great variety of routines and workloads as well as legal and financial issues. We think that lack of employment stability, relative low salaries, and overloaded work may have some impact on their health. Surprisingly, we could not find a significant level of anxiety among them. One explanation could be that the applied scale was not sensitive enough for the diagnosis of anxiety.

In summary, we found a frequency of 2.2% of TSD among Brazilian musicians. The predisposing factors were male gender, long duration of studying and practicing, and to playing classical instead of popular music. Despite of 63% of the DMs had sought for medical attention, more than half of them have not been not previously diagnosed with dystonia. Guitar and brass family of instruments, including trombone and tuba, were the riskiest to induce dystonia in Brazilian music players. Finally, we could observe that TSD has some particularities in Brazilian musicians probably due to cultural and regional differences.

## Author Contributions

RM: participated on the conception and execution of the research project, wrote the first draft, and reviewed the final version of the manuscript. PA: participated on the conception and execution of research project, planned the statistical analysis, made contributions on the writing, and reviewed the final version of the manuscript. GB: participated on the organizing phase and the execution of the research project, made contributions on the writing, and reviewed the final version of the manuscript. HF: participated on the conception, organization, and execution of research project, made contributions on the writing, and reviewed the final version of the manuscript.

## Conflict of Interest Statement

The authors declare that the research was conducted in the absence of any commercial or financial relationships that could be construed as a potential conflict of interest.
